# Sustainable concrete production through the integration of waste foundry sand, fly ash, silica fume and metakaolin

**DOI:** 10.1038/s41598-025-13277-9

**Published:** 2025-07-28

**Authors:** Tariq Ali, Muhammad Zeeshan Qureshi, Inamullah Inam, Nabil Ben Kahla, Hawreen Ahmed, Ali Ajwad, Muhammad Adnan

**Affiliations:** 1 Department of Civil Engineering, Swedish College of Engineering and Technology, Wah, 47080 Pakistan; 2 Department of Civil Engineering, University of Engineering and Technology, Taxila, Pakistan; 3 Department of Civil Engineering, Engineering Faculty, Laghman University, Mehtarlam, Afghanistan; 4 Civil Engineering Department, College of Engineering, King Khalid University, Abha, Saudi Arabia; 5 Center for Engineering and Technology Innovations, King Khalid University, 61421 Abha, Saudi Arabia; 6 Department of Highway and Bridge Engineering, Technical Engineering College, Erbil Polytechnic University, Erbil, 44001 Iraq; 7 Department of Civil Engineering, University of Management and Technology, Lahore, Pakistan

**Keywords:** Waste foundry sand, Silica fume, Strength, Durability, Metakaolin, Civil engineering, Engineering

## Abstract

This exploratory study investigates the use of waste foundry sand (WFS), combined with supplement cementitious materials (SCMs), in concrete production. The Preliminary Investigation is based on three groups, where the first group studies different percentages of natural sand replacement with WFS (i.e. 5%, 10%, 15% and 20%), the second group analyses the addition of 5% silica fume with WFS, and the third group observes 10% metakaolin inclusion with WFS. The studied parameters include density, nondestructive test (UPV), compressive and tensile strength, acid resistance, and environmental benefit analysis. According to the results, the compressive strength of the concrete mix is enhanced by 17% by adding 20% WFS and 5% silica fume, and this value increases by 23% when adding an additional 10% metakaolin. Furthermore, the use of 20% WFS leads to a 3.27% decrease in the cost of concrete as compared to the control mix, with a decrease of 2.1% and 5.1% for silica and metakaolin-containing mixes, respectively.

## Introduction

The economic development of a country is directly related to stability and growth in the construction industry, where concrete play a vital role, as it is the most utilized material globally. Internationally, concrete is used at a rate of 20–35 billion tones per year and this makes it the second most used product after water^1^. Concrete is the predominant construction material on a global scale, with an annual usage of 20–35 billion tons. The gradual increase in demand for this product can be due to its notable advantages, including its exceptional strength and durability, cost-effectiveness, flexibility in terms of shape and size, and resistance to water^2^. Interaction between governments and society is vital to offer economically and environmentally viable solutions. Concrete is produced using limited natural resources, which leads to environmental damage. Sustainable growth is crucial for the building industry, which utilizes many natural resources^3^. To utilize and get adequate sustainable disposal of waste, several countries have found a technique to incorporate solid waste into materials for construction^4,5^. The depletion of natural resources can be mitigated by incorporating waste materials or recycled industrial waste into concrete. This technique not only addresses disposal challenges but also presents a chance to explore alternative methods for conserving natural resources.

WFS (Waste Foundry Sand) is a potential material that poses comprehensive examination as a potential replacement for fine aggregates in concrete^6^. WFS is a secondary byproduct derived from casting ferrous and non-ferrous metals^7–10^. Among these industries, it is observed that ferrous foundries generate the largest quantity of sand. In recent years, there has been a significant increase in the utilization of foundry sand, an industrial waste material, for various construction projects^6,11,12^. Over an extended period of time, the utilization of WFS for landfilling has demonstrated its effectiveness. However, the rising expenses associated with waste disposal have presented substantial challenges to the adoption of landfilling techniques. The United States has over 3000 foundries, which utilize around 100 million tons of sand annually for their production operations. Consequently, a significant amount of waste foundry sand, ranging from 6 to 10 million metric tons, is dumped each year and deposited in landfills^13,14^. Foundry Sand has 85–95% pure silica sand, 2–5% water, 7–10% bentonite, and around 5% charcoal^15–18^.

The utilization of discarded foundry sand in the construction industry offers numerous advantages, which are otherwise major environmental concerns. One of the primary challenges foundries encounter is collecting and managing stockpiled waste foundry sand (WFS), necessitating substantial storage space. The presence of stored waste foundry sand poses a potential risk to the environment’s sustainability. This is mostly due to the significant leaching of toxic metals through the waste foundry sand, which has been seen to occur at a high rate^19^. Hence, it is crucial for foundries to develop innovative approaches to effectively reuse accumulated waste foundry sand to align with the concepts of a sustainable economy. This is necessary to prevent the stacked waste foundry sand from surpassing storage limits, which has the potential to impede production processes^20^. Furthermore, the tough restrictions and guidelines regarding trash disposal strengthen the industries’ endeavors and dedication to enhancing their rates of waste re-utilization or recycling, contributing to achieving the zero-waste objective. At now, landfilling is not regarded as the optimal solution. It is discouraged within a global context that prioritizes concepts such as the circular economy and promoting environmental sustainability, aiming to achieve zero waste^21^. Several foundries are grappling with significant penalties associated with waste disposal in landfills. This practice has become financially burdensome and negatively impacts their profit margins^22^.

The construction industry is experiencing significant growth and expansion, encompassing innovative methodologies to enhance efficiency and convenience in on-site operations. The sand subjected to the casting process in different industries can be reused several times. However, once it reaches a point where it is not for reuse, it is classified as “Waste foundry sand” and is then taken from the foundry for disposal. Using WFS as a partial substitute for the fine aggregate within concrete results in cost-effective, lightweight, and high-strength concrete. Based on its composition, it can be categorized into two classifications: (green foundry sand), Which is composed of silica sand to the level of 85–95%, Binding agent bentonite clay at a concentration of 4–10%, and 2–10% pulverized coal (to improve the appearance of the cast surface), or (chemical foundry sand). Has silica content between 93% and 99.9% and chemical-based 1–3% binding agents^23^.

Potential uses for waste foundry sand include the following areas: dams, construction of barrier layers, flowable fills, road construction, agriculture, specifically soil reinforcement and amendments, the manufacture of hot-mix asphalt, manufacturing of Portland cement, production of mortars, traction material for snow and ice, vitrifying of hazardous substances, smelting; manufacturing of rock wool, and production of fiberglass. This analysis utilized a small sample of studies that only recently began collecting data on WFS utilization. Large-scale implementation of the WFS is possible in many areas of the building industry, including geophysics and roadway construction. When the expenses of transporting the reused sand do not exceed those of transporting the initial form of sand, using the WFS becomes cost-effective^24^.

Supplementary cementitious materials (SCMs) play a pivotal role in advancing modern concrete production practices by addressing key challenges and optimizing performance. SCMs, such as fly ash, metakaolin, and silica fume, are integral components that offer multiple benefits to concrete formulations. Firstly, their integration enhances the overall durability of concrete structures by effectively mitigating the detrimental effects of weathering, chemical attack, and abrasion^25–29^. By reducing permeability and pore connectivity, SCMs contribute to improved resistance against degradation mechanisms like alkali-silica reaction and sulfate attack, thereby extending the service life of constructed assets^30,31^. Secondly, the use of SCMs promotes superior strength gain behaviour in concrete, facilitating enhanced mechanical performance over time^18,23,32^. This research explores a comprehensive investigation into the impact of SCMs on various concrete properties, emphasizing their eco-friendly characteristics and sustainability attributes^33^.

This research highlights the effectiveness of supplementary cementitious materials (SCMs) like fly ash, metakaolin, and silica fume in enhancing concrete performance and reducing environmental impact. These materials improve concrete strength, durability, and pore structure through their pozzolanic reactivity and high content of amorphous silicon dioxide. By incorporating SCMs, the study demonstrates a sustainable approach to optimizing concrete performance, aligning with principles of green construction practices.

## Novelty of the research

Mineral admixture is frequently employed in numerous studies due to its advantageous pozzolanic characteristics^34–36^. Most investigations in the field of concrete materials include binary blends^25,37–40^, where each material possesses distinct properties, and the addition of a certain material enhances only that particular property. There is limited literature available regarding ternary blends. When two materials with distinct qualities are combined into a single substance (ternary mix), the resulting product combines all the attributes of both materials. Ternary blends are better than binary mixes^41–43^. Therefore, this study aims to incorporate three distinct pozzolanic materials (ASTM C618 ), each possessing unique properties. Silica fume enhances the bond between aggregate and paste, while fly ash improves later strength. On the other hand, metakaolin is renowned for its contribution to early strength in concrete. Consequently, this investigation represents a novel exploration of the relationship between concrete strength and durability. This study will contribute vital insights to the existing literature.

Concrete materials are becoming more expensive, and this has an effect on the depletion of natural resources, thus, there is a growing need for low-cost, long-term solutions. The use of WFS as a partial substitute for fine aggregate in concrete is the focus of this research. The study encompasses a comprehensive assessment of concrete’s mechanical properties, specifically focusing on compressive and tensile strength. This exploratory evaluation involves the incorporation of waste foundry sand along with various SCMs to gauge their impact on the concrete’s strength characteristics. Additionally, the research delves into the durability aspects of a ternary blend, particularly emphasizing its resistance to water absorption and acid exposure. Furthermore, the investigation extends its scope to analyze the economic advantages and environmental implications of utilizing locally sourced foundry sand as a concrete component. This multifaceted study aims to provide valuable insights into enhancing concrete materials’ performance, sustainability, and cost-effectiveness in construction practices.

## Methodology

**Fig. 1:**
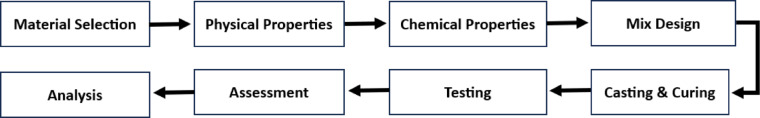
Flow chart of methodology.

The research investigations are systematically designed to conduct a comprehensive and detailed assessment of the behavior of foundry sand concerning extra supplementary materials (fly ash, silica fume, and metakaolin). The initial step involves selecting materials based on the requirements of the study investigation. Subsequently, these materials undergo a comprehensive examination, whereby their chemical and physical qualities are evaluated following international standards. The mix design is carried out per the ACI procedures, considering the characteristics of the selected materials.Concrete Mix Design is influenced by characteristics such as workability, compression strength, aggregate size, durability and grading. The mix design is primarily determined by strength, with the water-to-cement (w/c) ratio representing one of the key parameters in the mixed design. The rate of concrete’s compressive strength development is influenced by the type of cement used. The selection of cement type relies on the desired strength and other specific requirements. The trial mixes are subsequently cast based on the mix design, and the desired slump is reached by trial testing. Mixing is conducted in a large mixer, and adequate mixing is accomplished by allowing the mixer enough time to rotate and blend the ingredients uniformly. Concrete mixing takes 1 min if mixer is not overfilled and runs at an adequate speed. Most concrete mixers make usable concrete in 1–2 min. The cast material is placed in water for 28 days for the curing procedure. The rational behind 28 days curing, testing over decades has shown concrete reaches 99% strength after 28 days. The 28-day compressive strength standard is accepted by Portland Cement Association and ASTM International. The evaluation of compressive and tensile strength was conducted according to ASTM C-39 and ASTM C-496. The examination and analysis of UPV (ASTM C-597) and acid attack (ASTM C-1898) were conducted. Following the curing period, the material is assessed and analyzed. The flow chart of the project methodology is given below in Fig. 1.

### Materials

The Ordinary Portland Cement of Bestway Cement factory of Pakistan was utilized in this study, which fulfilled the requirements of ASTM C-150^42^. The chemical and physical properties of OPC were listed in Table 1, while locally available Lawrencepur sand was used for the fine aggregate. Easily located Coarse aggregate was made from crushed angular material from Margalla with a maximum particle size of 19 mm. Coarse and fine aggregates met ASTM C-33 requirements.The physical properties of aggregates are mentioned in Table 2.Fly ash (F Class) which is easily locally available were used in this study. In the current investigation, waste foundry sand were sourced from Islamabad steel mills pakistan. The bulk density and fineness modulus of foundry sand were less than those of natural sand. Table 3 provides the chemical composition of waste foundry sand.Table 4 presents the the physical and chemical properties of FA(fly ash), SF(silica fume) and MK (metakoalin).

**Table 1:** Chemical properties and physical properties of OPC.

Properties (physical)		Properties (chemical)	
Parameters	Outcomes	Elements	Percentage (%)
Consistency	30%	SiO_2_	21
Initial setting time	50 min	MgO	1.05
Final setting time	254 min	Al_2_O_3_	5.30
Sp. gravity	3.10	Cao	62
Specific surface	319 m^2^/kg	K_2_O	1.01
–	–	Na_2_O	0.23

**Table 2:** Coarse aggregate and fine aggregate physical Properties.

Parameters	Aggregate (coarse)	Aggregate (fine)	WFS
Fineness modulus	–	2.59	2.57
Sp. gravity	2.65	2.60	2.53
Unit weight (kg/m^3^)	1598	1715	1451
Water absorption (%)	0.71	0.641	1.45

**Table 3:** Waste foundry sand chemical composition.

Elements	Percentage (%)	Requirements (American foudry society (AFS), 1991)
SiO_2_	87.10	87.9%
MgO	0.26	0.30%
Al_2_O_3_	4.67	4.70%
Cao	0.92	0.14%min
K_2_O	0.02	–
Na_2_O	0.03	–
LOI	4.39	5.15%max
Sulfates	0.04	0.09%

**Table 4:** Chemical properties and physical properties of FA, MK, SF.

Properties (physical)		Properties (chemical)	
Parameters	Values	Elements	Percentage (%)
Fly ash			
Fineness	2500–6000 cm^2^/g	SiO_2_	40–60
Particle size	10–100 micron	MgO	1–5
Bulk density	0.9–1.2 g/cm^3^	Al_2_O_3_	20–30
Sp. gravity	2.1–2.8	Cao	5–15
–	–	Fe_2_o_3_	5–20
–	–	Na_2_O	0.5–1.5
Metakoalin			
Fineness	5000–8000 cm^2^/g	SiO_2_	50–55
Particle size	< 2 micron	K_2_O	< 1
Bulk density	0.5–0.7 g/cm^3^	Al_2_O_3_	35–45
Sp. gravity	2.5–2.7	Cao	< 1
–	–	Fe_2_o_3_	1–2
–	–	Na_2_O	< 0.5
Silica fume			
Fineness	15,000–30,000 cm^2^/g	SiO_2_	85–98
Particle size	0.1–1 micron	K_2_O	0.2–1
Bulk density	0.3–0.6 g/cm^3^	Al_2_O_3_	0.5–2
Sp. gravity	2.2–2.3	Cao	0.5–1
–	–	Fe_2_o_3_	0.5–2
–	–	Na_2_O	0.2–1

### Rational behind the selection of material

The use of waste foundry sand (WFS) in the production of concrete is a potential replacement for natural sand because it has the most advantages in terms of being an industrial raw material compared to other industrial byproducts.WFS possesses high silica sand and there is a large availability of it at very low cost, which makes it a best material for high volume concrete production. WFS is free of harmful elements compared to the other industrial by-products like copper slag and steel slag which comprises heavy metals and other toxic elements, making WFS environment friendly for construction. In addition to this advantage, WFS was found to enhance the mechanical properties of concrete, including tensile and compressive strength, which reveals the potential uses of WFS for environmentally friendly concrete production^8–10^. The chosen values for percentage replacements of waste foundry sand (WFS) in concrete mixes were 5%, 10%, 15% and 20% which were derived as a combination of preliminary studies and literature benchmarks. Previous studies showed that a small percentage of WFS (5–10%) display a considerable enhancement in concrete properties while maintaining acceptable workability and durability in concrete^7,44^. The following dosage rates for fly ash, metakaolin, and silica fume were examined in this study, based on the literature survey, properties of materials and initial trial mixtures. The materials used in this work were ordinary Portland cement, fly ash replacing at a rate of 50 kg/m³ which provides an economical and durability solutions^45,46^, metakaolin at a percentage of 10% because of its early strength^33,47^, and silica fume at 5% of the total volume due to bonding and compressive strength enhancement^48,49^.These rates were established by trial mixes to attain desirable workability, strength and durability with regard to test groups.

### Concrete mixes composition

**Fig. 2:**
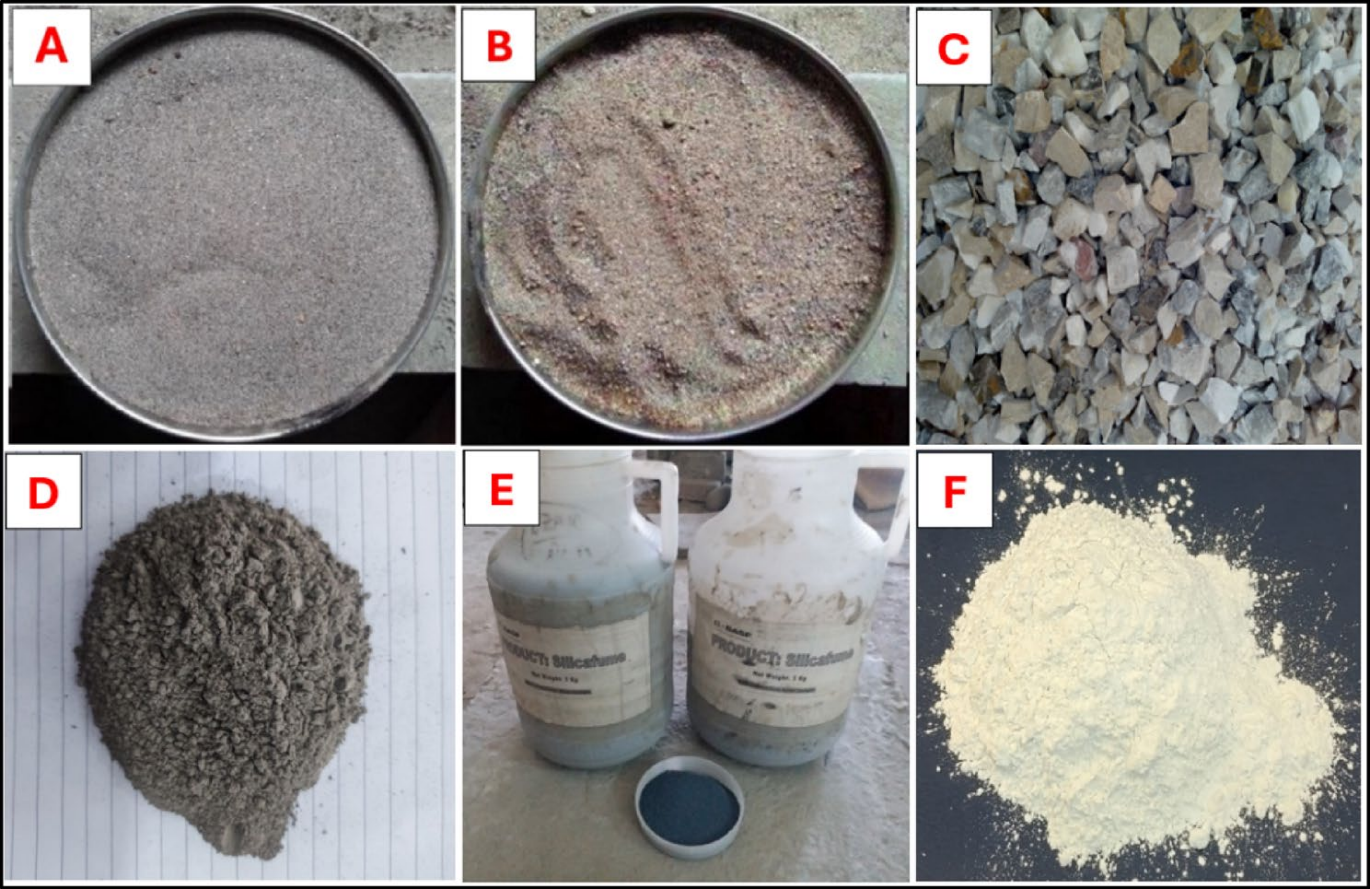
Material used for casting (**A**); Natural sand, (**B**); WFS, (**C**) Coarse aggregate, (**D**) Fly ash, (**E**) Silica fume, (**F**) Metakaolin.

For this experiment, 15 concrete mixes were made with different foundry sand (FS) and constant values of Fly ash percentages to examine the performance combined with 10% metakaolin and 5% silica fume-incorporated concrete. As per the research findings the WFS is mostly in a range from 0 to 25% as replacement of sand in concrete^44,50^. The interval was maintained consistently, and various pozzolanas were incorporated into the concrete mixture to improve its mechanical qualities. The concrete blends were produced by replacing a percentage of the Natural Sand (NS) with different percentages (5%, 10%, 15%, and 20%) of foundry sand (FS). Three groups were tested and evaluated in this research; each group comprised five subgroups. In all these fifteen groups, constant-value cement (350 kg/m^3^) and fly ash (50 kg/m^3^) were used. The details of subgroups (SG) were M0-S0-F0, M0-S0-F5, M0-S0-F10, M0-S0-F15, and M0-S0-F20. M stands for metakaolin, S stands for silica, F stands for foundry sand, and numerical subscript shows their content percentage. The slump for all the mixes was kept in the range of 70–90 mm by adjusting the water-to-binder (w/b) ratio to 0.5. The WFS had finer particles, but the presence of fly ash (50 kg/m^2^) and superplasticizer minimized excessive water demand. Previous studies^14,44^ stated that workability was not significantly influenced by WFS replacements ≤ 20% when supplementary cementitious materials (SCMs) such as fly ash were present. But due to the increased surface area, higher WFS contents (> 30%) may necessitate the use of water-reducing admixtures^51^. Table 5 provides mix design of the each combinations. Concrete mixes were cast in the lab within controlled environmental conditions. Cement was fully mixed with the fly ash to achieve uniform concrete mixes. Then, using a laboratory concrete mixer, all the constituents of concrete (binders, fine aggregates, and coarse aggregates) were combined and mixed for a minimum of two minutes. Estimated water was gently added and mixed for two more minutes to get concrete with appropriate workability. The freshly mixed concrete was placed in a tray and slump-tested to determine workability. After that, the concrete mix was poured into molds, compressed, and cured for 24 h. After 24 h, the concrete samples were removed from the molds and soaked in normal water at 27 °C until testing age.The experiments were carried out thrice and the mean values were used for all cases to minimize on errors and enhance reliability. The materials used in this investigation is shown in Fig. 2 .

**Table 5:** Composition of mixtures.

Mixes	Cement (kg/m^3^)	Fly ash (kg/m^3^)	Silica fume (kg/m^3^)	Metakaolin (kg/m^3^)	WFS (%)	Aggregate (kg/m^3^)		W/C ratio
						Fine	Coarse	
M0-S0-F0	350	50	0	0	0	650	1150	0.5
M0-S0-F5	350	50	0	0	32.5	617.5	1150	0.5
M0-S0-F10	350	50	0	0	65	585	1150	0.5
M0-S0-F15	350	50	0	0	97.5	552.5	1150	0.5
M0-S0-F20	350	50	0	0	130	520	1150	0.5
M0-S5-F0	332.5	50	17.5	0	0	650	1150	0.5
M0-S5-F5	332.5	50	17.5	0	32.5	617.5	1150	0.5
M0-S5-F10	332.5	50	17.5	0	65	585	1150	0.5
M0-S5-F15	332.5	50	17.5	0	97.5	552.5	1150	0.5
M0-S5-F20	332.5	50	17.5	0	130	520	1150	0.5
M10-S0-F0	315	50	0	35	0	650	1150	0.5
M10-S0-F5	315	50	0	35	32.5	617.5	1150	0.5
M10-S0-F10	315	50	0	35	65	585	1150	0.5
M10-S0-F15	315	50	0	35	97.5	552.5	1150	0.5
M10-S0-F20	315	50	0	35	130	520	1150	0.5

The mixture of fly ash, metakaolin, silica fume, and waste foundry sand within the concrete matrix encapsulates a holistic approach to sustainability and socio-economic viability. By minimizing carbon emissions, conserving energy and resources, and offering potential long-term economic advantages, your creation embodies the principles of sustainable development in construction, charting a path towards a more ecologically harmonious and economically robust built environment.

## Results and discussion

### Compressive strength

**Fig. 3:**
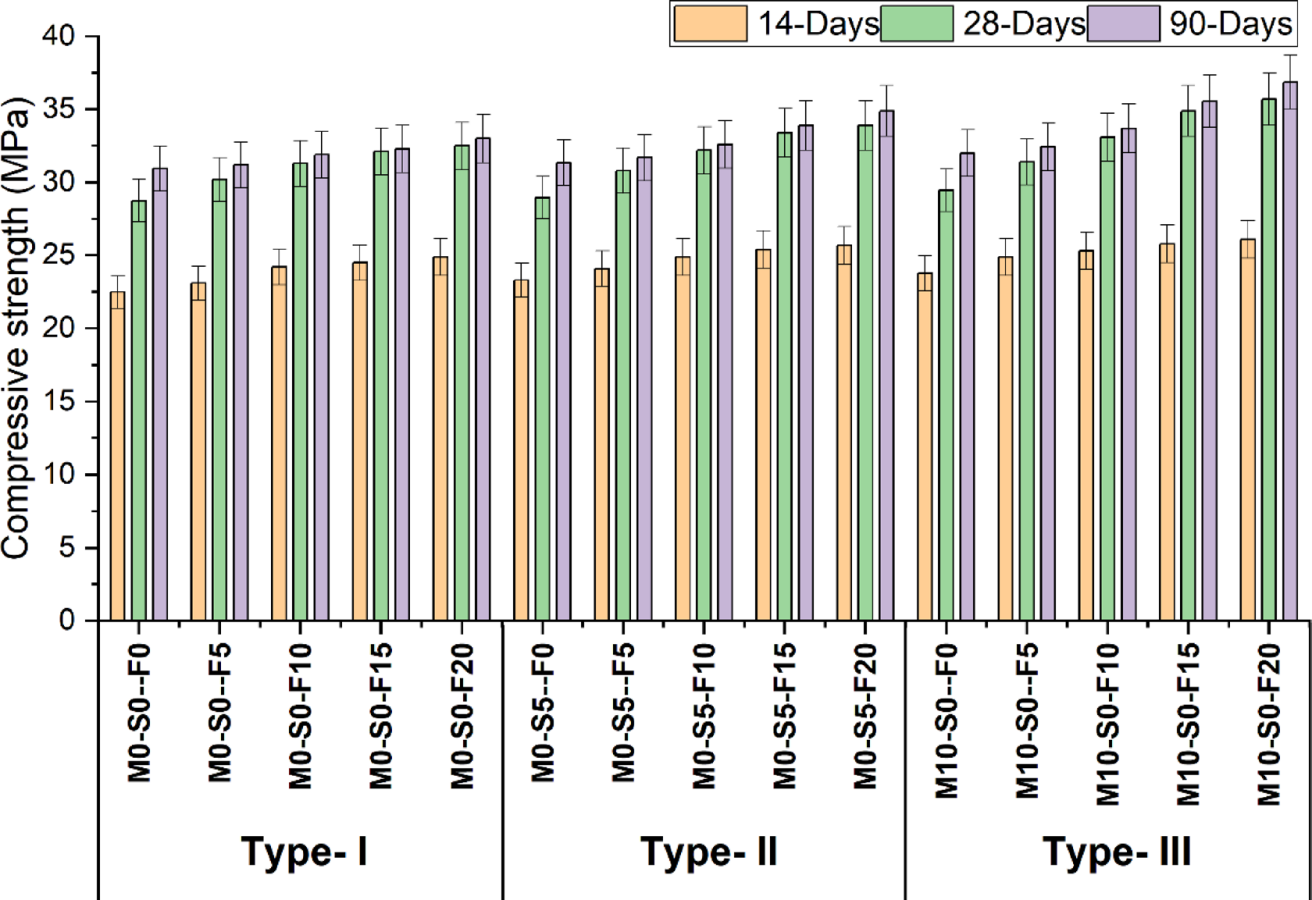
Compressive strength test result at 14, 28 and 90 days.

Figure 3 illustrates the effectiveness of concrete with varying quantities of WFS, replacing 10% metakaolin and 5% silica fume while maintaining a consistent proportion of fly ash. The test is performed on cylinder size 150 mm x 300 m according to ASTM C39. In the Type-1 mix, it has been shown that the strength increases with the addition of foundry sand. Specifically, the highest compressive strength values are obtained when 20% of the regular natural sand is replaced with foundry sand. The strength measured at 14, 28, and 90 days and are found to be 24.9 MPa, 32.5 MPa, and 33 MPa, respectively.

Incorporating foundry sand has enhanced strength in Type-2 mixtures (containing 5% silica fume). The optimal values of strength is also achieved by substituting 20% of the control mix even in the case of 5% silica fume utilization.The results evaluated at three different time intervals: 14, 28, and 90 days. The corresponding measurements were 25.7 MPa, 33.9 MPa, and 34.9 MPa, respectively. Moreover, a similar pattern is observed in the context of Type-3 mixtures, where the strength is influenced by adding 10% metakaolin in conjunction with foundry sand. The achievement of the most promising strength values is accomplished through the replacement of 20% of the control mix with foundry sand. The measurements were undertaken at three periods, specifically 14, 28, and 90 days, resulting in the collected data. The readings obtained were determined to be 26.1 MPa, 35.7 MPa, and 36.9 MPa, respectively. Table 6 shows the Discriptive statistics of compressive strength.

According to^52^ analysis, the compression strength of concrete partially substituted with WFS is greater than that of standard concrete. This is because WFS contains finer granules, which serve as an excellent filler and make for a denser concrete mixture. The void filling of granular aggregates reduces the amount of air pockets within the solidified concrete matrix. Silica may have helped form CSH gel^53^. The chemical reaction between the SiO_2_ in the WFS and the CH produced during cement hydration results in CSH. CSH enhances the binding properties of concrete, increasing its durability^52^.

**Fig. 4:**
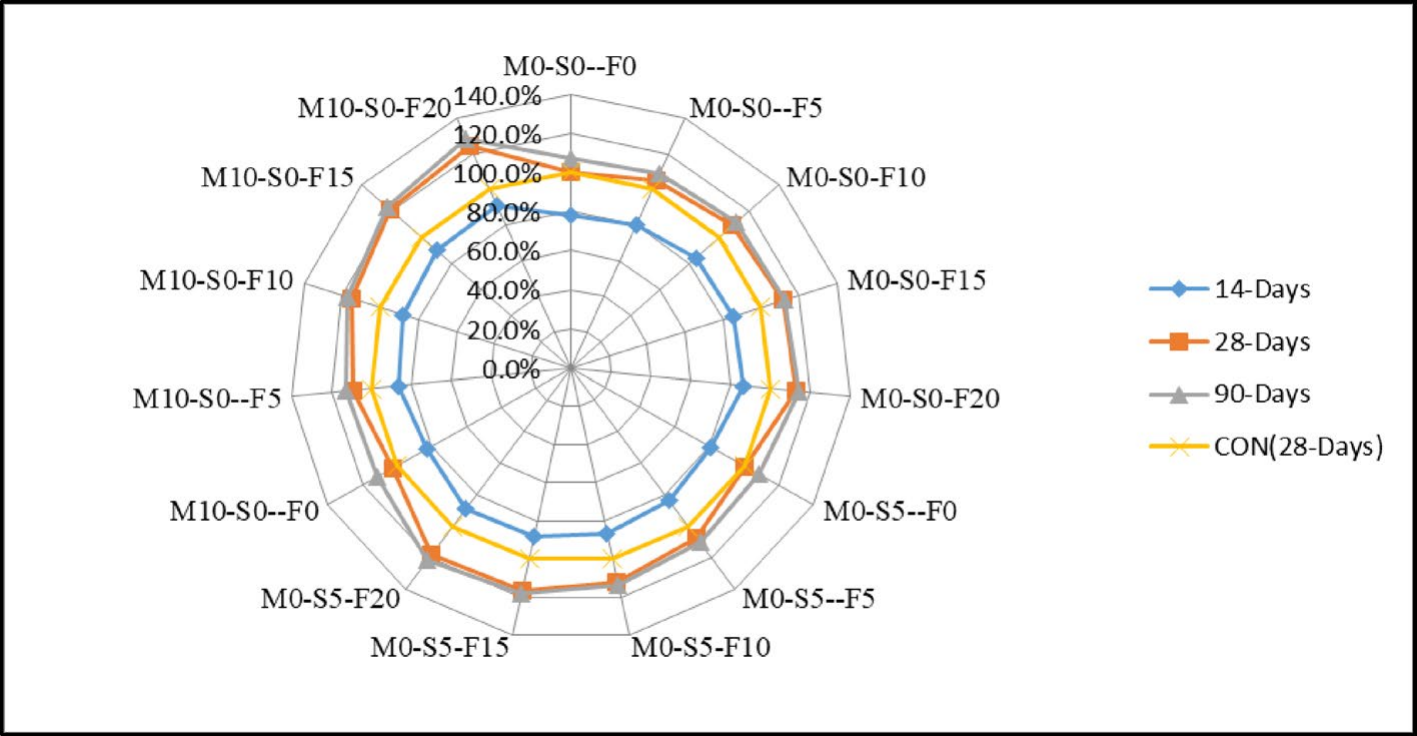
Comparison of compressive strength with each mix at CON (28 days).

Similarly, the radar chart in Fig. 4 indicates that control M0-S0-F0 is used as a point of reference, rising to 8.8% having 10% WFS. Comparatively, the 20% WFS (waste foundry sand) exhibits a 13% increase at 28 days, whereas the control mix with 10% WFS shows a 4% increase. Similarly, the 20% WFS demonstrates a 9% increase at 90 days. Furthermore, the graph demonstrates that the biggest increase is observed for 10% metakaolin, with a rise of 15.1% for 10% WFS and 24.1% for 20% WFS, respectively. The graph indicates that the 28-day compressive strength is enhanced when using various amounts of WFS + Fly ash (5%, 10%, 15%, and 20%). In the control mix containing 5% WFS, the strength was measured to be 30.2 MPa. This strength increased to 30.8 MPa when 5% Silica fume was added. Similarly, when 10% of metakaolin was replaced, the strength increased from 29.5 MPa to 31.4 MPa, including 5% WFS.

**Fig. 5:**
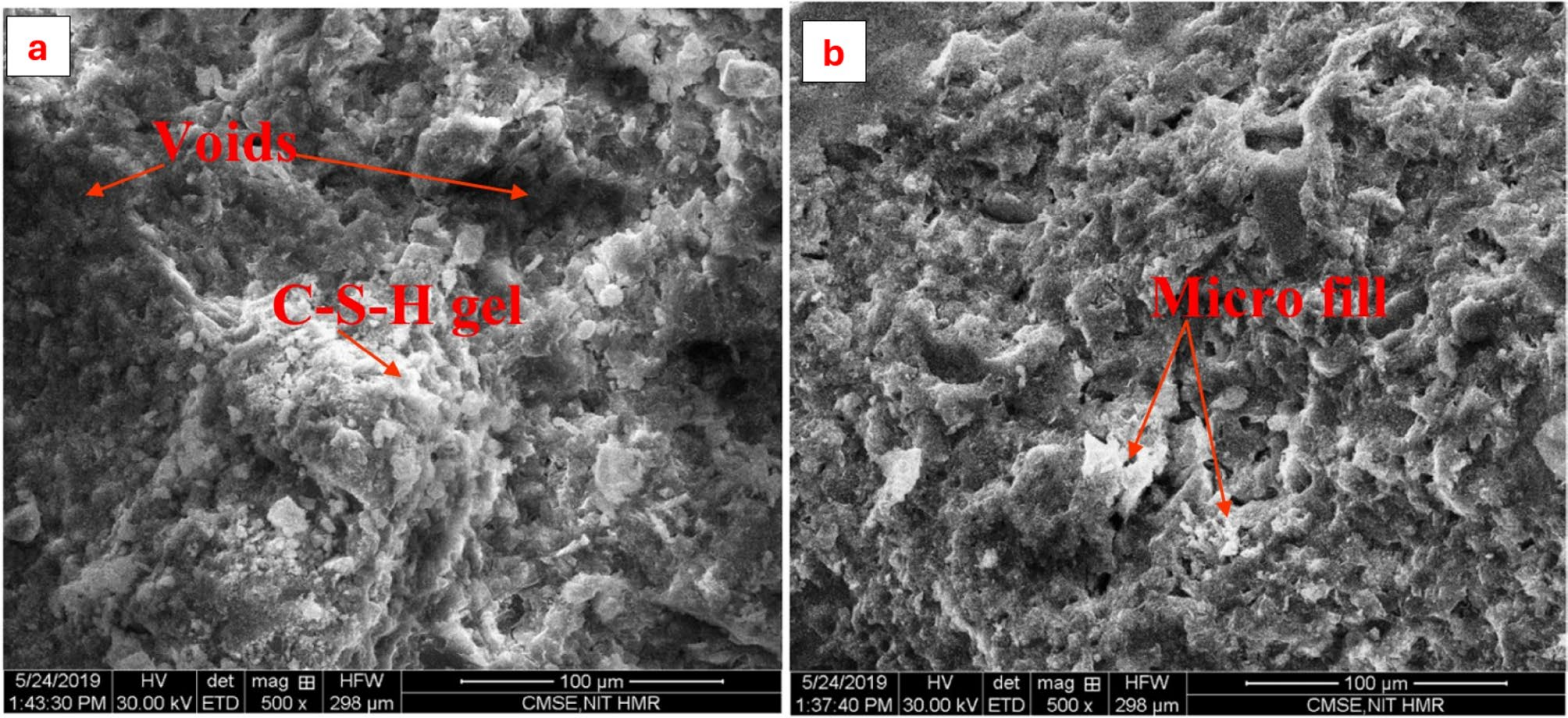
SEM analysis (**a**) Normal concrete (**b**) Waste foundry sand^40^.

The scanning electron microscope (SEM) images (Fig. 5a and b) depict the influence of replacing waste foundry sand on the microstructure of regular concrete and concrete made with waste foundry sand. The compact structure observed in Fig. 5a, characterized by dense C–S–H gel and minimum voids, leads to increased concrete strength when compared to the control specimen. However, Fig. 5b illustrates a less compact C–S–H gel in the concrete made from construction waste coarse aggregate. This suggests that the strength of this concrete is enhanced compared to regular concrete, but it is still lower than the strength of concrete that has been substituted with waste foundry sand^40^.

**Table 6:** Descriptive statistics of compressive strength.

Curing days	Mean	Standard deviation	SE of mean
14-Days	24.56	1.05	0.27
28-Days	31.91	2.08	0.53
90-Days	32.95	1.72	0.44

### Split tensile strength

**Fig. 6:**
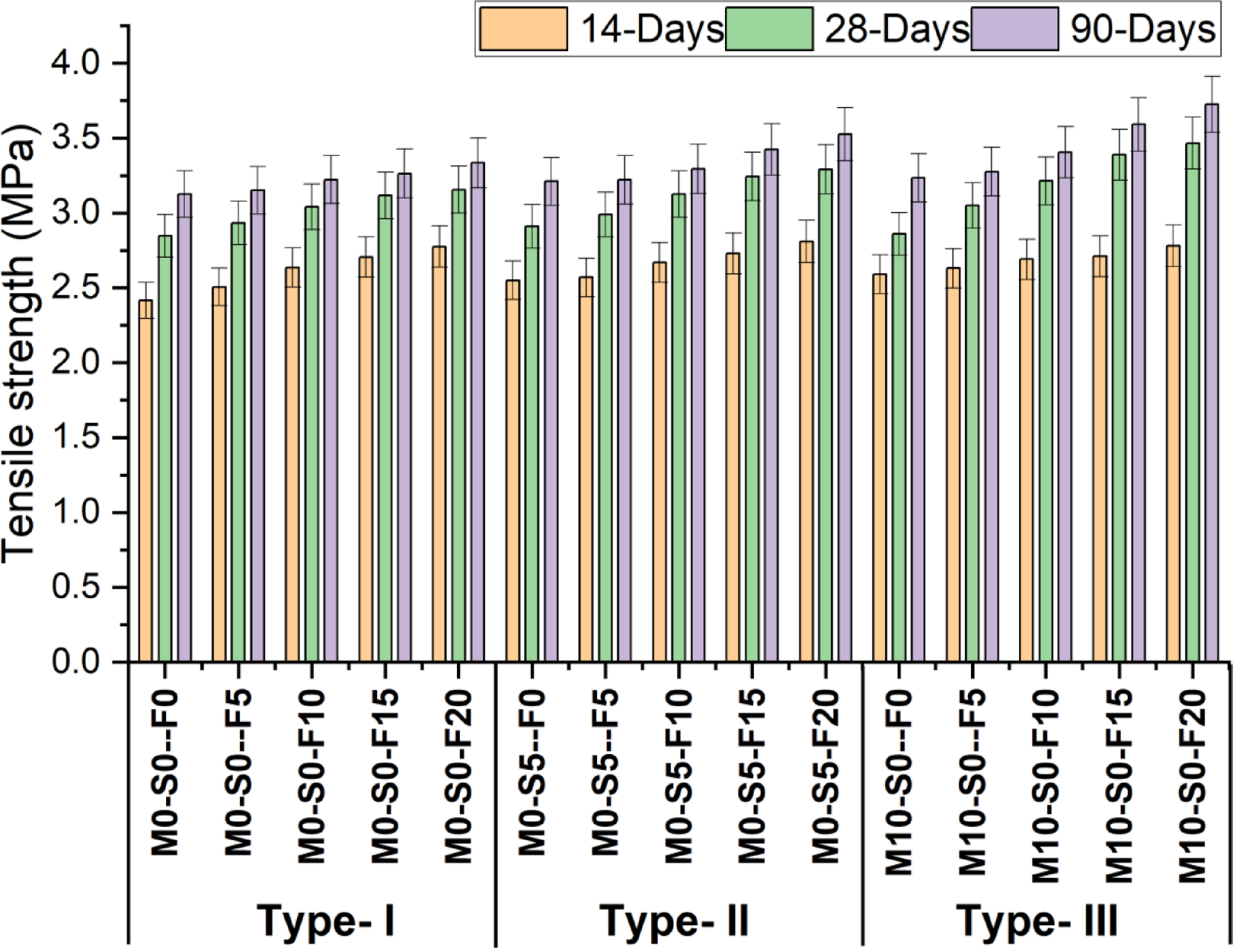
Tensile strength test result at 14, 28 and 90 days.

Figure 6 demonstrates the variations in the tensile strength of concrete when varied quantities of waste foundry sand (WFS) are used, with the substitution of metakaolin (10%) and silica fume (5%) while maintaining a consistent proportion of fly ash.The test is performed on cylinder size 150 mm x 300 m according to ASTM C496. The Type-1 mix has demonstrated a positive correlation between the incorporation of foundry sand and the enhancement of tensile strength. The optimal strength values are achieved by substituting 20% of the regular natural sand with foundry sand. The aforementioned values are obtained by measurements conducted at three different time intervals: 14, 28, and 90 days. The corresponding values recorded are 2.7 MPa, 3.1 MPa, and 3.3 MPa, respectively.

Furthermore, a similar trend may be observed in the case of Type-3 mixtures, where the inclusion of 10% metakaolin in combination with foundry sand impacts the tensile strength. The most positive strength values are achieved by substituting 20% of the control mixture with foundry sand. The measurements were conducted at three distinct intervals, namely 14, 28, and 90 days. The obtained readings were 2.7 MPa, 3.4 MPa, and 3.7 MPa, respectively. The descriptive statistics of tensile strength are shown in Table 7.

Similar findings revealed^54^ that WFS partially replaced natural sand at different percentages (10, 20, 30, 40, 50 and 60%). Among the many substitutions tested, it was found that the 30% substitution demonstrated the highest tensile strength. In contrast to our research findings, some studies have shown a decline in the tensile strength and durability of concrete due to increased foundry sand content^55^. Research conducted^51^ has also observed a decline in tensile strength due to utilizing foundry sand. This can be attributed to the porosity and low density of concrete caused by small dust particles in the foundry sand.The “Small Dust Particles” under the foundry sand includes residues such as silica dust, and clay particles from the sand casting process. These particles normally are below 75 microns in size and are attributed to make up the sand mortar of the concrete.

**Fig. 7:**
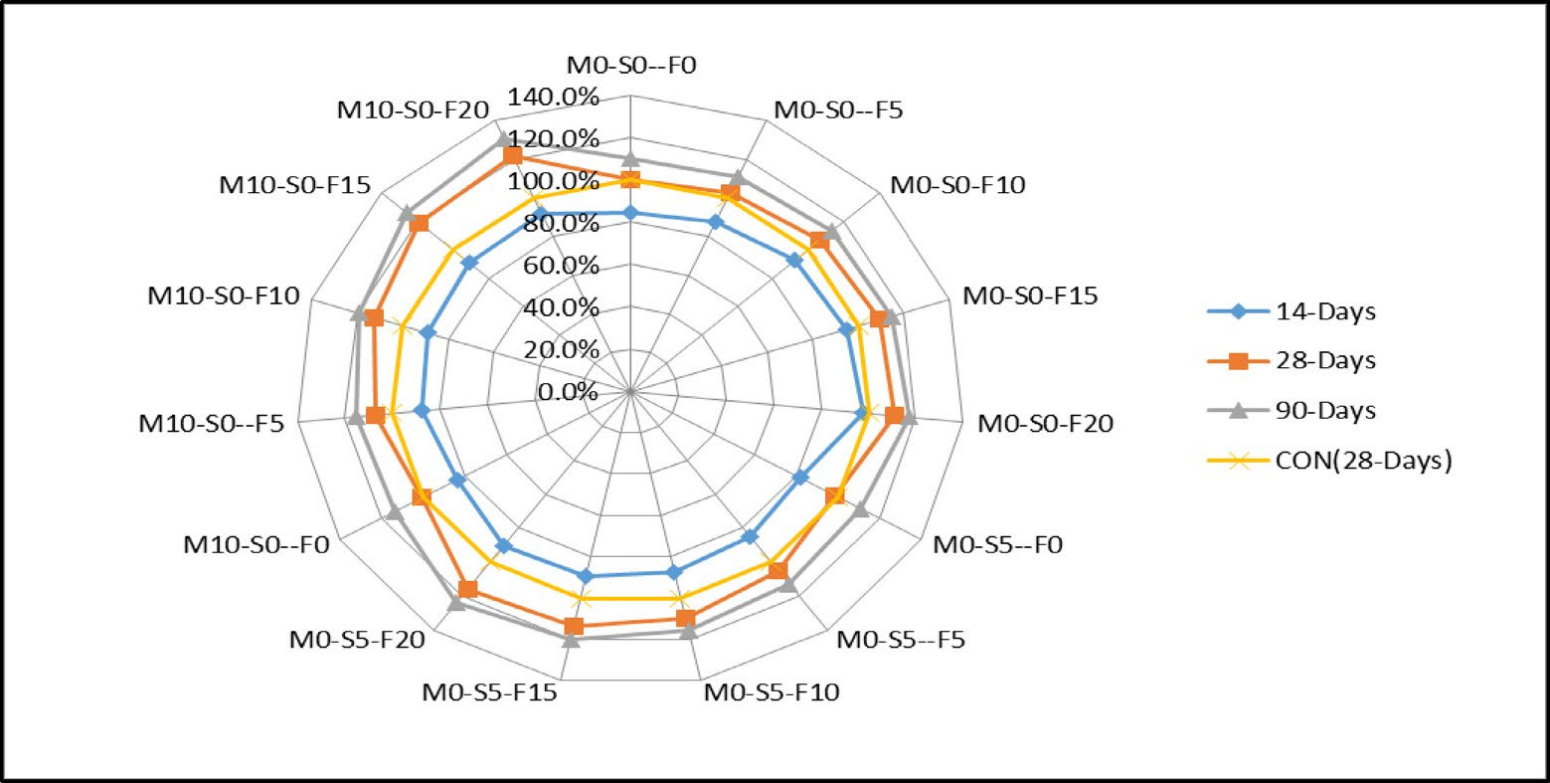
Comparison of tensile strength with each mix at CON (28 days).

The radar chart in Fig. 7 demonstrates that control M0-S0-F0 serves as a baseline, with an increase of 6.7% for 10% WFS and 10.8% for 20% WFS at 28 days. The control mix shows a 4% increase for 10% WFS and a 9% increase for 20% WFS at 90 days. Furthermore, the graph demonstrates that the largest increase in tensile strength is observed with 10% metakaolin, with a value of 12.9% for 10% WFS and 21.7% for 20% WFS. The graph indicates that various percentages of WFS (5%, 10%, 15%, and 20%) enhance the 28-day tensile strength. The control mix containing 5% WFS exhibited a split tensile strength of 2.93 MPa. This strength increased to 2.99 MPa when 5% Silica fume was added. Similarly, when 10% of metakaolin was replaced, the split tensile strength increased from 2.93 MPa to 3.05 MPa for 5% WFS. Concrete’s theoretical compressive strength is eight times greater than its tensile strength. This suggests a constant connection between concrete’s compressive and tensile strengths.

**Table 7:** Descriptive statistics of tensile strength.

Curing days	Mean	Standard deviation	SE of mean
14-Days	2.65	0.11	0.028
28-Days	3.10	0.18	0.048
90-Days	3.33	0.17	0.043

### Density

**Fig. 8:**
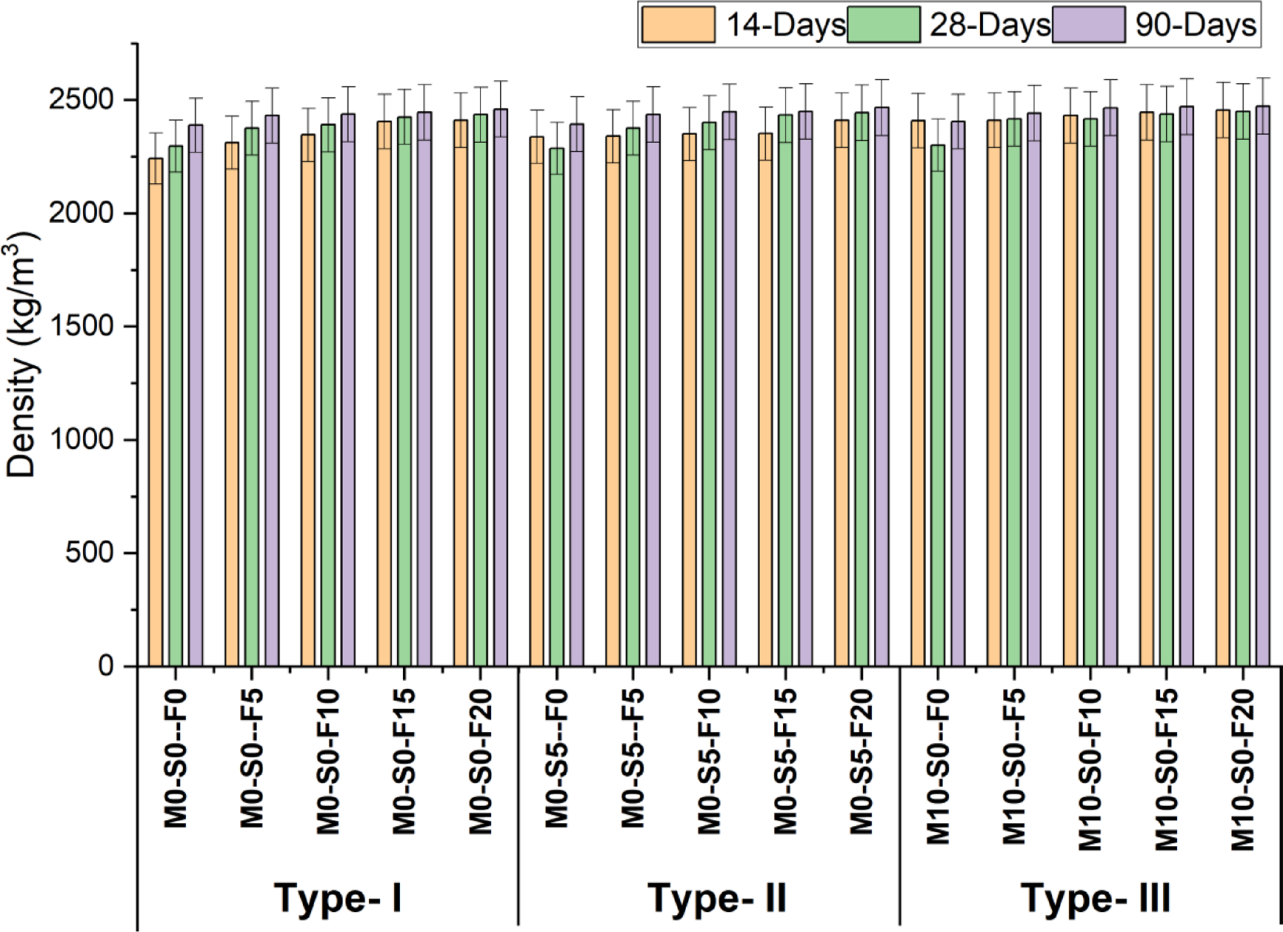
Density test results at 14, 28 and 90 days.

Figure 8 depicts the changes in the dry density for all 3 types of mixes with varying percentages of foundry sand. It is clear from the figure that the density increases in all three groups due to the micro-filling effect of foundry sand, which causes more dense concrete. The highest density is achieved with 20% foundry sand whose value in type-1, 2 and 3 mix is 2411 kg/m^3^, 2420 kg/m^3^ and 2456 kg/m^3^. The density of concrete is closely related to its compressive strength, as the presence of open voids and air pockets determines it. Table 8 shows the descriptive statistics of density.Several studies have documented a reduction in the density of concrete when including foundry sand due to the creation of air pockets within the particles of the material^50^. At the same time the initial density of the control mix of fresh concrete is about equal to the density of concrete achieved when WFS is substituted for regular sand at a range of ten to thirty%^14^.

**Table 8:** Descriptive statistics of density.

Curing days	Mean	Standard deviation	SE of mean
14-Days	2377.92	57.64	14.88
28-Days	2393.13	55.39	14.30
90-Days	2441.73	26.76	6.90

### Ultrasonic pulse velocity (UPV)

**Fig. 9:**
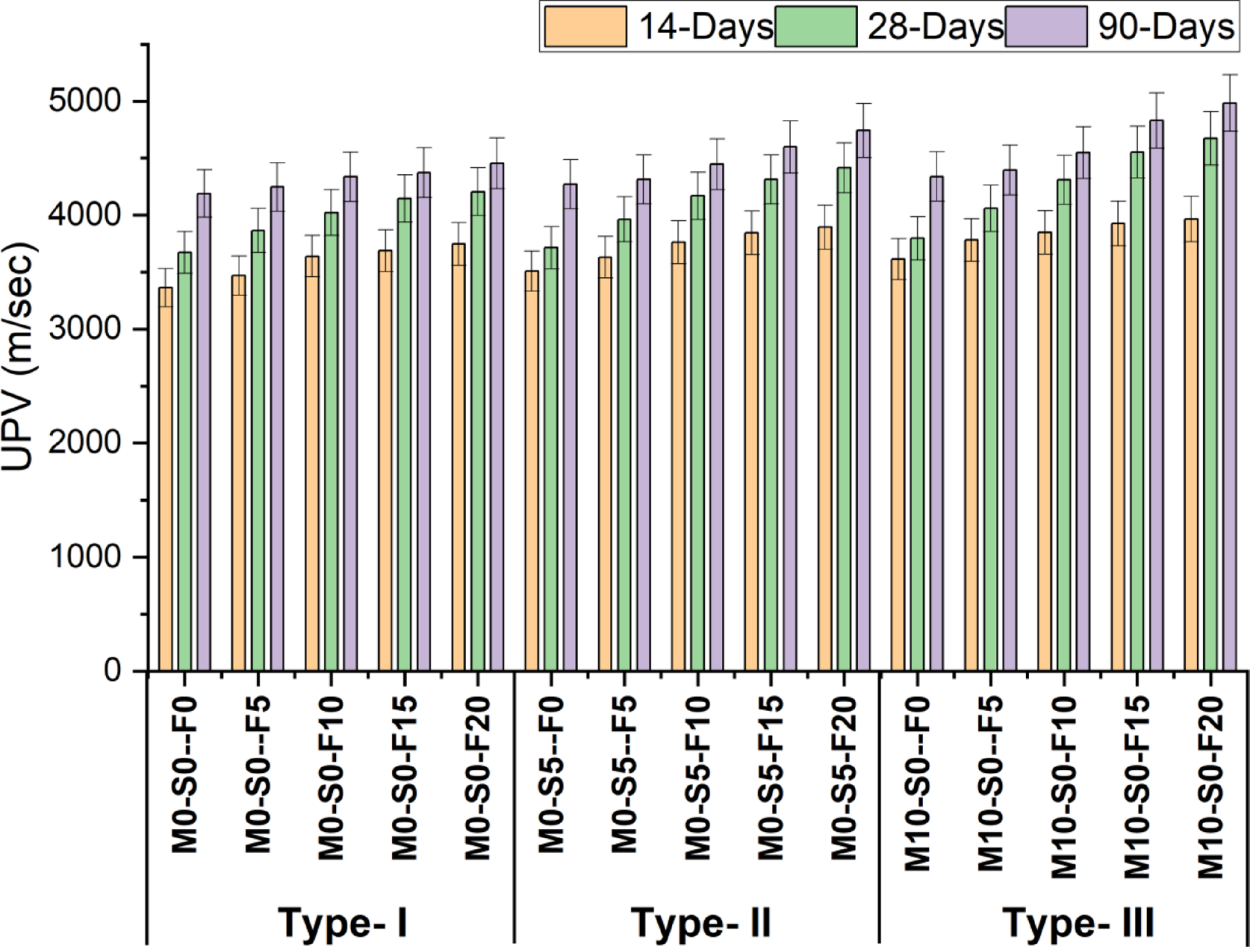
UPV test result at 14, 28 and 90 days.

Figure 9 illustrates the variations in the UPV for all three mixtures as the percentages of foundry sand increase the UPV value increase. The Figure demonstrates that the UPV value increases in all three groups due to the micro-filling effect of foundry sand, leading to denser concrete. The maximum UPV (ultrasonic pulse velocity) is obtained using 20% foundry sand, resulting in values of 3747 m/s, 3893 m/s, and 3964 m/s for type-1, type-2, and type-3 mixtures, respectively. the ultrasonic pulse velocity depends on the density and elasticity of the material, which is affected by the compressive strength of concrete. The improvement in the UPV indicates better density and elastic property of the concrete thus resulting into the better compressive strength. Thus UPV is a useful and effective test in analyzing changes in material heterogeneity and density, which makes it a valuable for evaluating the quality, uniformity, and performance of concrete used in structural applications. The descriptive statistics are shown in Table 9.

**Table 9:** Descriptive statistics of UPV.

Curing days	Mean	Standard deviation	SE of mean
14-Days	3711.9	175.44	45.29
28-Days	4124.90	297.23	76.74
90-Days	4471.21	229.13	59.16

### Acid attack

**Fig. 10:**
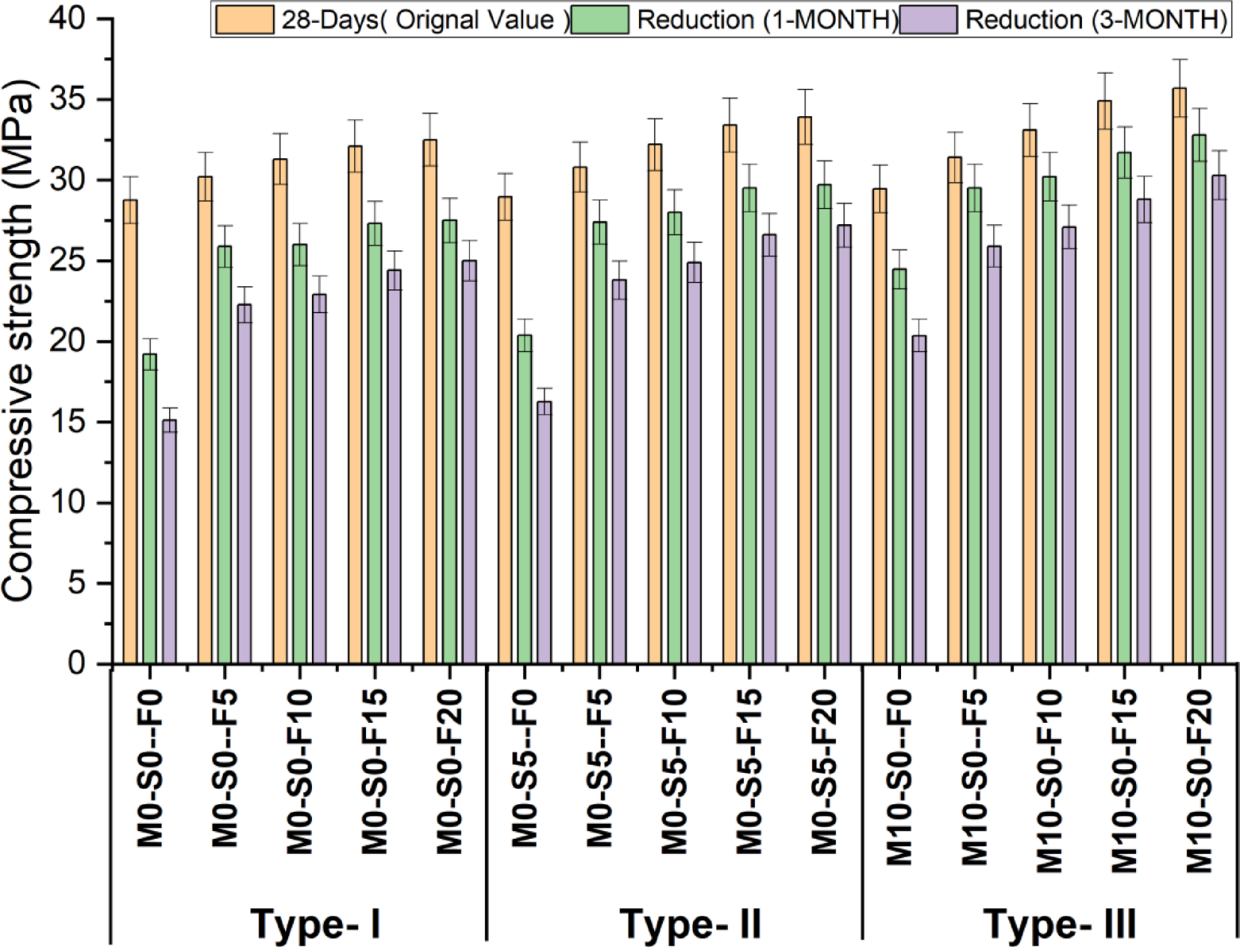
Comprehensive strength in 5% HCL environment.

Figure 10 shows the performance against the acid exposure for all three types of mixtures as the percentages of foundry sand increase. The test is carried out in accordance to ASTM C267 standrad. The type-1 mix containing fly ash exhibited 40% higher resistance than the control mix (without supplementary cementitious material), with 20% foundry sand. The high strength and density of the material can be due to the pozzolanic features of fly ash, which promote the formation of a significant amount of C–S–H gel^49^. The type-2 mix, including silica fume, exhibits a 45% greater resistance than the control mix. The enhanced performance of this material is attributed to the characteristic of silica fume, which enhances the adhesive strength between the aggregate and paste, decreases the calcium content, and reduces the concrete porosity; as a result, concrete becomes more impermeable and durable^48,56–58^. Furthermore, in the case of the type-3 mix, the resistance is 53% more than the control mix. When MK replaces some of the Portland cement in a mixture, less tricalcium aluminate hydrate is required to create the cement paste matrix. The second process involves consuming some of the calcium hydroxides produced during cement hydration via a pozzolanic reaction involving the MK and the calcium hydroxide. As a result, MK concrete will have less of the expansive gypsum produced by the reactivity of calcium hydroxide than ordinary concrete. Although the pozzolanic reaction’s secondary C-S-H is less thick than the original C–S–H gel, it does a good job of filling and segmenting otherwise enormous capillary gaps into smaller, discontinuous ones. As a result, concrete loses some of its permeability overall^47,59^. The descriptive statistics are shown in Table 10.

**Table 10:** Descriptive statistics of acid attack.

Curing days	Mean	Standard deviation	SE of mean
14-Days	31.91	2.08	0.53
28-Days	27.30	3.77	0.97
90-Days	24.06	4.23	1.09

## Cost analysis

Aggregates constitute a significant component of concrete, and the cost depends entirely on the aggregates’ price. This research replaces fine aggregate with the WFS, a waste material derived from ferrous and nonferrous metal casting. This substitution is expected to significantly lower costs. The cost analysis of concrete for a volume of one cubic meter is conducted by comparing the expenses of control concrete with those of concrete that includes recycled foundry sand. Table 11 shows the prices of each material.

**Table 11:** Price of materials.

Sr. No	Material description	Price (Rs/kg)
1	OPC (Fauji)	30
2	Metakaolin	16
3	Fly ash	13
4	Silica fume	50
5	Fine aggregate (Lawrenspur)	8
6	Coarse aggregate (Margalla)	10
5	WFS	1

The findings indicate that using WFS in the concrete mixture results in a more cost-effective solution than the control concrete. A 20% substitution of waste foundry sand (WFS) results in a 3.27% reduction in cost for one cubic meter of concrete, shown in Table 12, while the highest reduction is found for that of 10% metakaolin with 20% WFS, the cost reduction is 5.03%. All the analysis for the cost is reported in local currency (Pakistani rupee).

**Table 12:** Sand (Replaced with foundry sand) cost analysis for one cubic meter.

Mixes	Cement (kg/m^3^)	Fly ash (kg/m^3^)	Silica fume (kg/m^3^)	Metakaolin (kg/m^3^)	WFS (%)	Aggregate (kg/m^3^)		Total cost	Percentage reduction
						Fine	Coarse		
M0-S0-F0	10,500	650	0	0	0	5200	11,500	27,850	0.00
M0-S0-F5	10,500	650	0	0	32.5	4940	11,500	27,622.5	0.82
M0-S0-F10	10,500	650	0	0	65	4680	11,500	27,395	1.63
M0-S0-F15	10,500	650	0	0	97.5	4420	11,500	27,167.5	2.45
M0-S0-F20	10,500	650	0	0	130	4160	11,500	26,940	3.27
M0-S5-F0	9975	650	875	0	0	5200	11,500	28,200	-1.26
M0-S5-F5	9975	650	875	0	32.5	4940	11,500	27,972.5	-0.44
M0-S5-F10	9975	650	875	0	65	4680	11,500	27,745	0.38
M0-S5-F15	9975	650	875	0	97.5	4420	11,500	27,517.5	1.19
M0-S5-F20	9975	650	875	0	130	4160	11,500	27,290	2.01
M10-S0-F0	9450	650	0	560	0	5200	11,500	27,360	1.76
M10-S0-F5	9450	650	0	560	32.5	4940	11,500	27,132.5	2.58
M10-S0-F10	9450	650	0	560	65	4680	11,500	26,905	3.39
M10-S0-F15	9450	650	0	560	97.5	4420	11,500	26,677.5	4.21
M10-S0-F20	9450	650	0	560	130	4160	11,500	26,450	5.03

## Limitattion and future work

This research examined the performance of waste foundry sand (WFS) individually and combinations SCMs materials. The research has some limitations. Significant limitations are that it lacks long-term durability tests (e.g., freeze-thaw tests, sulfate attack, carbonation), it is locally available materials-based, and it lacks a full environmental study (e.g., life cycle assessment). To advance these findings, the next phases of this work will focus on:

- Long-term durability testing under exposure conditions to assess actual usage performance.
- Environmental monitoring with LCA, weighing CO₂ reduction versus processing energy and resource consumption.
- Scale-up at industrial levels through pilot testing, cost estimation, and stakeholder interactions.
- Material heterogeneity through experimentation using WFS originating from different industrial processes and regions.

## Conclusion

- The incorporation of WFS improves the compressive strength of the concrete which is an essential criterion for using it as a construction material. It was observed that substantiate for the mix of M0-S0-F20 mix (20% WFS), 17.9% increase in compressive strength at 28 days and on replacing 10% metakaolin this increase was risen to 24.1%. Including a 20% proportion of WFS into the mix increases the tensile strength as well.
- Although UPV values and density values are improved with WFS inclusion, UPV values and density values of WFS substitution have remarkable improvements with values increasing up to 20% WFS substitution levels. All three concrete mixes that were tested show that as the percentage of WFS increased, the acid resistance performance improved.
- WFS has great potential to reduce the costs of raw materials and to support sustainable waste disposal options in concrete. The economic impact is visible by the savings of 3.27% cost per cubic meter obtained when 20% WFS replaces natural sand. The cost of optimal mix of concrete with 2% silica fume was decreased 2.01% and that of 10% metakaolin was decreased 5.03%.

## Data Availability

The data is available from the corresponding author upon request.
